# Multidisciplinary Care of a Vertebral Fracture in a Patient with Hematopoietic Stem Cell Transplant: Safety Appropriateness in Interventional Pain Management and Rehabilitation Considerations

**DOI:** 10.3390/healthcare10030497

**Published:** 2022-03-08

**Authors:** Vinicius Tieppo Francio, Brandon Barndt, Usman Latif, Sarah M. Eickmeyer

**Affiliations:** 1 Department of Rehabilitation Medicine, The University of Kansas Medical Center, Kansas City, KS 66160, USA; seickmeyer@kumc.edu; 2 Department of Physical Medicine and Rehabilitation, Temple University/MossRehab, Philadelphia, PA 19140, USA; brandonbarndtdo@gmail.com; 3 Department of Anesthesiology, The University of Kansas Medical Center, Kansas City, KS 66160, USA; ulatif@kumc.edu

**Keywords:** vertebral fracture, pain management, rehabilitation, kyophoplasty, hematopoietic stem cell transplant

## Abstract

Bone loss leading to fragility fracture is a highly prevalent late effect in hematopoietic stem-cell transplant patients, who are affected 8–9 times more than the general population, particularly for vertebral compression fractures. Spinal interventions such as lumbar epidural steroid injections and vertebral augmentation may be helpful for providing pain relief and improved function, quality of life and return to ambulation. However, interventional procedures should be approached with caution in these patients. Our study found that there is a paucity of scientific studies addressing the risks of spinal injections in these patients and there is no absolute recommendation specific to spinal injections in patients receiving immunosuppressive agents or who have a history of solid organ or hematopoietic stem cell transplant. It is imperative to consider proper timing of the intervention to minimize risks while optimizing the benefits of the intervention combined with a well-defined post-transplant rehabilitation plan. Moreover, the decision to proceed with spinal interventions should be done case by case and with caution. Therefore, this article reports the case of a multidisciplinary treatment for a vertebral compression fracture in a patient with a hematopoietic stem-cell transplant, in particular discussing safety appropriateness in interventional pain management and rehabilitation considerations for this condition in this patient population.

## 1. Introduction

Bone loss leading to fragility fracture is a highly prevalent late effect in hematopoietic stem-cell transplant (HSCT) patients, who are 8–9 times more likely than the general population to be affected, primarily within 6 to 12 months post-transplant [1]. Fragility fractures usually imply a low-energy trauma etiology, such as lifting, bending, or falling from a standing height. Risk factors include intensive chemotherapy, total-body radiation, menopause, weight loss, and medications such as glucocorticoids used for treating graft-versus-host-disease (GVHD). The immunocompromised state after HSCT increases not only the risk of fragility fracture but also procedure-related infections. Furthermore, HSCT and post-HSCT medications increase the risk of anemia, thrombocytopenia, chronic pain, chronic fatigue, steroid myopathy and peripheral neuropathy [2,3,4]. Vertebral fractures (VFs) can cause debilitating pain, reduced quality of life (QoL), and increased morbidity and mortality [1,5,6].

HSCT can be very stressful on the body’s physiology and can leave patients with significant debility; therefore, supportive care measurements to improve QoL and overall survival are key components in post-transplant care. Physical rehabilitation aims to improve post-transplant musculoskeletal sequelae, such as pain, fatigue, and inflammation [7,8,9,10]. In this population, VFs are often asymptomatic, and there is usually minimal or no acute trauma history. Therefore, within the post-transplant phase, a HSCT patient who presents with new-onset back pain should be evaluated for VF. Pain is usually worse with weight-bearing and forward-flexion of the spine, and a neurological exam is often normal. In these situations, spinal radiographs may be obtained and may often be normal. Therefore, magnetic resonance image (MRI) may be the modality of choice to demonstrate an acute fracture with new focal vertebral body edema. Accurate diagnosis is essential for deciding the need for intervention treatments for pain control or vertebral body restoration to optimize the early return to mobility [6,7,8].

A spinal injection, such as a lumbar epidural steroid injection (ESI), can provide short-term relief from back pain and an improvement in function. However, an ESI may also exacerbate bone mineral density (BMD) loss [11]. Vertebral augmentation (VA) techniques have been shown to restore vertebral height, reduce pain and disability, and decrease mortality and morbidity in patients with vertebral fractures, so they may be potential options for selected HSCT patients [6,12,13,14,15,16]. Although these spinal interventions have been proven safe and effective in the general population, there is a paucity of studies discussing their use specifically in the HSCT population [17,18,19].

Therefore, this case report discusses the multidisciplinary treatment of a vertebral fracture in an HSCT patient, particularly emphasizing safety appropriateness in interventional pain management and rehabilitation treatment considerations.

## 2. Materials and Methods

A case report.

## 3. Results/Case Report

**Figure 1:**
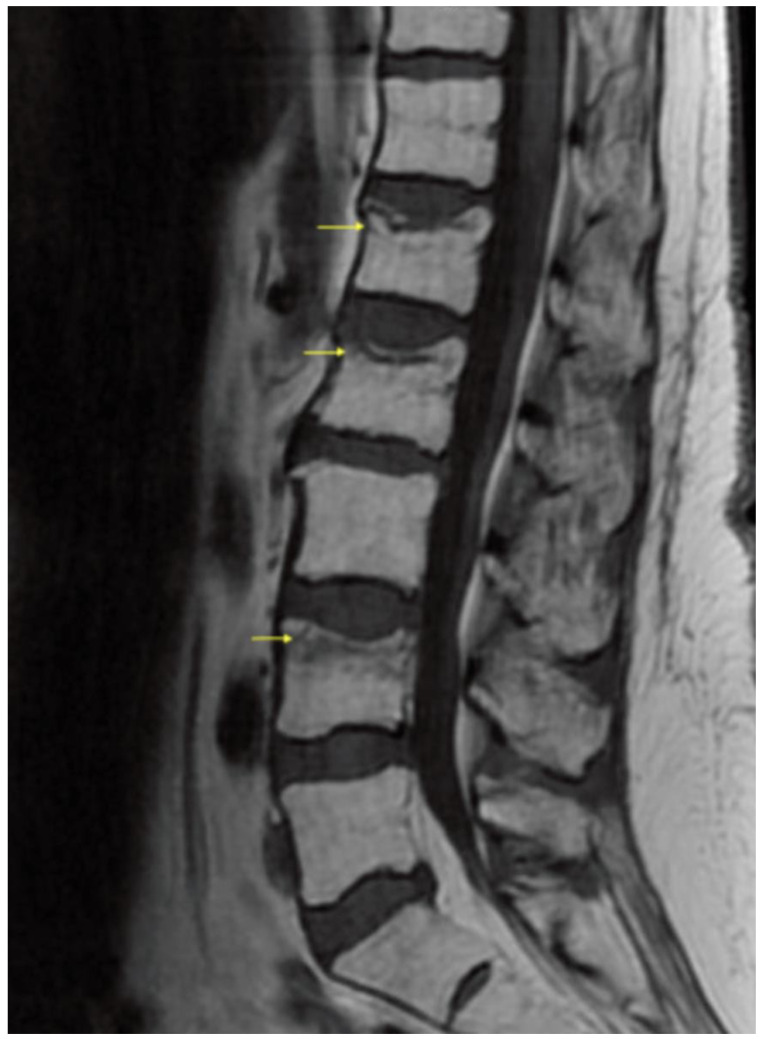
Lumbar spine magnetic resonance T1 image (MRI) with yellow arrows demonstrating superior endplate vertebral compression fractures of the L1, L2 and L4. In particular, the prominent bone marrow edema of the L4 vertebral endplate is consistent with an acute/subacute compression fracture.

**Figure 2:**
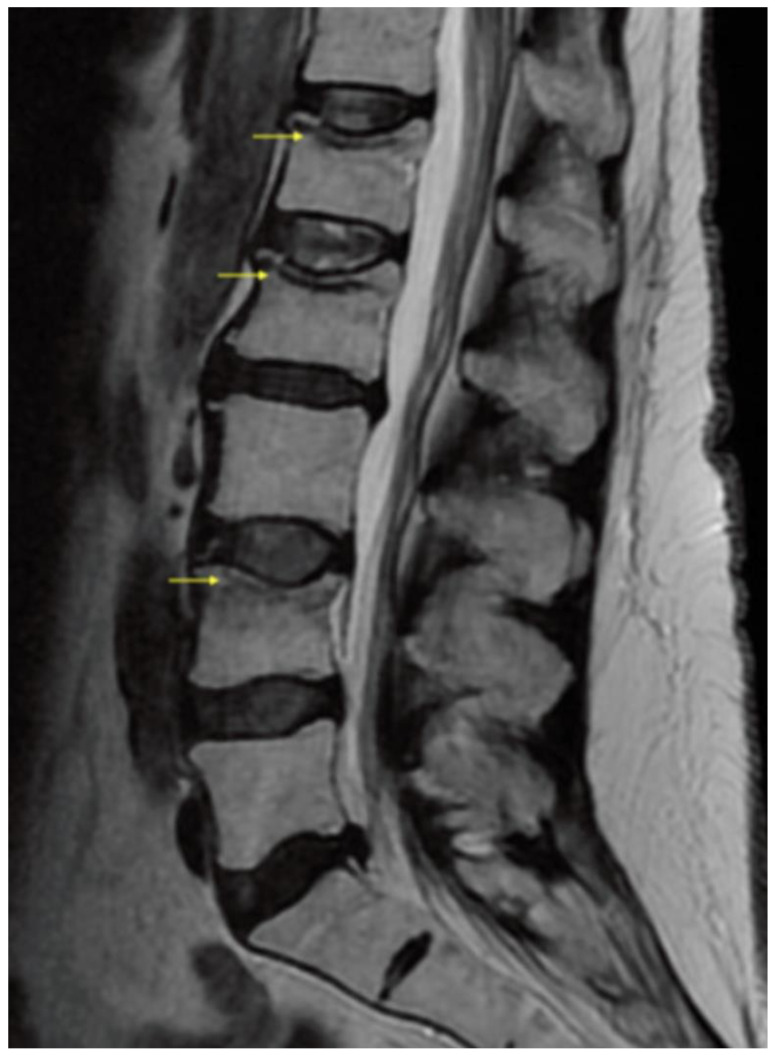
Lumbar spine magnetic resonance T2 image (MRI) with yellow arrows demonstrating superior endplate vertebral compression fractures of the L1, L2 and L4. There is mild postcontrast enhancement at the L1 and L2 compression fractures with minimal STIR hyperintensity edema and moderate postcontrast enhancement involving the superior L4 endplate.

A 58-year-old female with a medical history of acute myeloid leukemia post-HSCT, pancytopenia, steroid-induced myopathy, GVHD, chronic immunosuppression, and treatment with glucocorticosteroids, was transferred to the inpatient rehabilitation unit on day 90 post-transplant. Her hospital course was complicated by recurrent Gram negative and enterobacter cloacae complex bacteremia and atypical pneumonia. She made significant progress while in rehabilitation, until two-weeks into her course, when she developed acute onset low back pain (LBP) without radicular symptoms. Symptoms started after taking a shower during a supervised occupational therapy session. Specifically, no fall, trauma, or accident was reported by the patient or staff. The day after reaching forward in the shower, she reported a gradual onset of back pain localized in the thoracolumbar region and lumbar spine. In addition to her previous Tylenol and opioid analgesic therapy, she was prescribed ice, heat, cyclobenzaprine and a lidocaine patch. A physical examination revealed a limited spinal range of motion, bilateral paravertebral muscle tenderness to palpation without focal spinous process tenderness, no erythema, and no ecchymosis. There were no neurological deficits: the seated straight-leg raise test was negative, manual motor testing did not reveal any focal weakness, deep tendon reflexes were symmetrically equal, and the sensation was grossly intact. The pain persisted for a few days, without significant response to increased dosages of analgesic and adjunctive pain relief medications as one would expect with mechanical musculoskeletal back pain. Furthermore, the patient had a slight decline in function, and had more difficulty participating in therapies, and performing activities with the physical and occupational therapists, which prompted further evaluation with advanced diagnostic imaging. A lumbar spine magnetic resonance (MRI) scan was performed, which revealed superior endplate compression of L1, L2, and L4 vertebral bodies, with prominent bone marrow edema at the L4 level, which was consistent with an acute L4 VF, and old L1 and L2 VFs (Figure 1 and Figure 2). There was no significant retropulsion, and vertebral body height was reduced by less than 30%. The patient was diagnosed with a non-traumatic fragility fracture of the L4 vertebral body and re-admitted to the hematology service. Pain service was consulted to assess treatment options, and the decision was made to proceed with a lumbar ESI to facilitate a return to early mobility with improved pain control. The patient tolerated the procedure well without adverse events. It was recommended that she wear a thoracic lumbar sacral orthosis (TLSO) brace when out of bed for two weeks. The patient was readmitted the same day to the inpatient rehabilitation unit and continued her rehabilitation course uneventfully. She was eventually discharged home to her family with improved mobility and function, similar to baseline before the fragility fracture.

## 4. Discussion

### 4.1. Multidisciplinary Rehabilitation

Multidisciplinary rehabilitation in HSCT can be divided into pre-rehabilitation, post-rehabilitation, and survivorship. In particular, pre-transplant rehabilitation is important because it facilitates the maintenance of a minimum level of function focusing on cardiopulmonary function and improving muscle conditioning. This level of function can be assessed in pre-transplant patients using the baseline functional assessment tests: the 6 min walk, 50 foot fast walk, timed repeated sit-to-stand, forward reach, and grip/pinch strength [7,8]. This can also be used to identify any pre-transplant musculoskeletal impairment.

Post-transplant inpatient rehabilitation emphasizes reducing the symptomatic burden of the patient, as well as pain management and minimizing the risks of secondary complications such as pneumonia, pressure injury, and bowel/bladder dysfunction. Inpatient rehabilitation, particularly supervised exercise components, has also been shown to reduce fatigue in cancer survivors [7,8,9]. Additionally, it focuses on optimizing early mobility to prevent venous thrombosis, sarcopenia, and early bone loss. Post-transplant structured exercise programs have been shown to have a positive impact on survival [7,8,10]. Furthermore, during the post-transplant period, once the patient is medically stable and can be discharged from acute-care service, transfer to an inpatient rehabilitation setting may be possible if he or she is unable to be safely discharged home [7,8,9,10].

It is important to note that glucocorticosteroids are often used in post-transplant treatment, which is known to increase the risk of bone loss in a dose-dependent manner [7,8,20]. Therefore, in high-risk, post-HSCT patients it is important to begin preventative antiresorptive treatment, such as bisphosphonate therapy before, or as soon as possible after, transplant. Dual energy X-ray absorptiometry is recommended within 1 year of transplant. Yearly densitometry scans post-transplant are recommended. Vitamin D assessment is important as most HSCT patients are deficient; therefore, supplementation is critical, particularly within the 3 months post-HSCT [3,21]. Additionally, prevention of glucocorticoid-induced VF with calcium (1500 mg/day) and vitamin D supplementation (800 IU/day) is highly recommended. A trial of intranasal calcitonin for pain relief may be an option. A systematic review reported high-certainty evidence that bisphosphonates are beneficial for reducing the risk of VFs with data extending to 2 years, in particular with moderate-certainty evidence that these medications are beneficial for preventing and treating corticosteroid-induced bone loss at the lumbar spine and femoral neck, with little to no harm [22]. Similarly, a more recent meta-analysis revealed that bisphosphonate therapy is most likely to benefit postmenopausal women with osteoporosis who have a life expectancy greater than 12.4 months, but this may be a limiting factor in this population [23]. All patients with a high risk of corticosteroid induced bone loss should be counseled on lifestyle measures to maintain bone strength including nutrition and weight-bearing exercise. Pharmacological therapy should be considered for all patients at moderate to high risk of fracture because a favorable safety profile using oral bisphosphonates as the current mainstay of therapy showed evidence for preventing steroid-induced bone loss and reducing fracture risk [24].

In the ambulatory patient in the inpatient rehabilitation phase, at least 30 min of daily weight-bearing exercises is recommended to help maintain proximal muscle strength and function and to prevent steroid-induced myopathy. It also serves to decrease the risk of falls and secondary complications of immobility, such as pneumonia and thrombus venous embolism. Thoracolumbar bracing may be an option for reducing pain and preventing excessive forward flexion, particularly if there is an acute VF. Multi-modal exercise and nutritional programs after a fragility fracture of the spine have been shown to improve function and QoL and have been shown to offer improved morbidity and mortality [7,8,9,10,25].

### 4.2. Interventional Pain Management

Interventional spinal procedures should be approached with caution in HSCT and immunocompromised patients [26] because those receiving post-transplant glucocorticoids are at increased risk of bone loss and fragility fracture. Preventive and assessment measurement such as the Fracture Risk Assessment Tool (FRAX) has not been validated in the HSCT population [3].

In our case report, the decision to withhold VA and proceed with a single lumbar transforaminal epidural steroid (TFESI) injection bilaterally at the L4–5 level was made with several critical clinical considerations in mind. The patient was severely thrombocytopenic (platelets <20,000) two days prior to the procedure. Thrombocytopenia is a significant risk with potentially catastrophic effects when located nearby the spinal cord; therefore, we decided to minimize this risk by performing a low-risk intervention, in contrast to a significantly higher-risk intervention, such as vertebral augmentation. Furthermore, the goal of the intervention was to reduce pain and facilitate an early return to mobilization and rehabilitation therapies; therefore, the patient and the medical team opted for the TFESI, in contrast to the VA, which is a longer procedure entailing more tissue and instrument manipulation, more pain, and with the perceived increased risk of adverse events and complications. Caution should be taken when performing spinal procedures with a platelet count less than 80,000, including greater attention while performing the intervention and monitoring for catastrophic complications, such as epidural hematoma and nerve paralysis. Post-procedure vital signs and repetitive neurological examination, as well as written information to the patient on monitoring for complications should be given. Spinal procedures are usually contra-indicated when the platelet count is less than 50,000 [27]. In addition, the VF did not reduce vertebral body height by more than 30% as it was the superior endplate that was injured the most. VA is indicated when there is >30% vertebral body height loss [14]. Additionally, spinal epidural hematoma is a rare potential complication that may result in significant morbidity [28]. Two days prior to the procedure, the patient received numerous blood and platelet transfusions, and the intervention was performed with both hemoglobin and platelets above the recommended levels. She tolerated the procedure well without adverse events. In this particular population, the risk of post-procedure infections is also substantially higher [4,29]. While there have been numerous case reports of infections after a lumbar ESI, none of these had been related to an immunocompromised state, and most were associated with poor infection-control practices or an infected injectate [30]. To minimize procedure-related infection while still providing an appropriate clinical benefit, it was safer to proceed with a lumbar TFESI, a quicker, lower-risk intervention with minimal needle manipulation and less equipment-patient interaction compared to a VA, which is a longer, higher-risk, more elaborate procedure with multiple device exchanges involving needle entry from the skin into the pedicle and vertebral body, which potentially increases the risk of infection complications. Infection rates vary in the literature, but the VA has been found to have more than twice the relative risk compared to ESIs [31,32,33]. Particularly in a relatively immunocompromised patient, it is prudent to minimize risk of potentially life-threatening infection.

Our review did not find any studies published comparing the use of lumbar TFESI versus VA for the management of VF. This is not a surprise as the use of VA has been widely documented as both safe and effective in the management of VF in the general population, while the use of lumbar TFESIs has been well-documented in providing relief in patients suffering from persistent back pain and radiculopathy [14,19,34]. Although epidural steroid injections carry a much lower risk in contrast to vertebral augmentation, its use should be approached with caution, particularly in the HSCT population. A retrospective study reported that ESIs were associated with a significant decrease in bone mineral density, particularly with an increase in injection frequency and cumulative doses of steroids in the epidural space. However, the risk of subsequent fracture was quite small, likely not outweighing the potential benefits from pain relief and improvement in function from a single injection as in the present case [11].

## 5. Conclusions

There is a well-documented increased risk of bone loss and VF fracture in patients post-HSCT. Multimodal exercise and nutritional programs have been shown to improve function, QoL and reduce morbidity and mortality from VF fractures. Interventional pain procedures for these patients should be approached with caution, as commonly performed procedures in the general public, such as VA, may carry significant risks in this population due to an immunocompromised state often combined with pancytopenia. Appropriate platelet level and function must be accessed and confirmed since impairment may lead to potentially catastrophic consequences, such as spinal hematoma. Infection risks associated with a high-risk procedure, such as VA, could be significantly more dangerous for an immunocompromised patient; as such, other minimally invasive options may be a safer alternative for providing short-term pain relief and a faster return to mobilization and function, but there is limited data discussing safety considerations of interventional pain management options in this population. While there are no clear recommendations, it is essential to consider proper intervention timing to minimize risks while optimizing the intervention benefits. The decision to proceed with treatment should be made with caution on a case-by-case basis. In this case, we decided to proceed with a lumbar ESI to facilitate return to early mobilization with improved pain control, and the patient was readmitted the same day to the inpatient rehabilitation unit and continued her rehabilitation course uneventfully until being discharged home with improved mobility and function, similar to baseline before the fragility fracture.
